# *Dirofilaria repens* Infection and Concomitant Meningoencephalitis

**DOI:** 10.3201/eid1511.090936

**Published:** 2009-11

**Authors:** Sven Poppert, Maike Hodapp, Andreas Krueger, Guido Hegasy, Wolf-Dirk Niesen, Winfried V. Kern, Egbert Tannich

**Affiliations:** Bernhard Nocht Institute for Tropical Medicine, Hamburg, Germany (S. Poppert, A. Krueger, G. Hegasy, E. Tannich); Albert-Ludwig-University, Freiburg, Germany (M. Hodapp, W.-D. Niesen, W.V. Kern)

**Keywords:** Dirofilariasis, Dirofilaria repens, microfilaria, meningoencephalitis, cutaneous nodule, parasites, dispatch

## Abstract

*Dirofilaria repens,* a filarial nematode of dogs and other carnivores, can accidentally infect humans. Clinical symptoms are usually restricted to a subcutaneous nodule containing a single infertile parasite. Here, we report a case of *D. repens* infection with a subcutaneous gravid worm and the patient’s concomitant meningoencephalitis and aphasia.

*Dirofilaria repens* is a filarial nematode that affects dogs and other carnivores. Infections have been reported from various regions of the world, mainly from Europe, Africa, and Asia. As with other filaria species, mosquitoes transmit infectious microfilariae, which develop into fertile macrofilariae in their definitive host. Humans may become infected as aberrant hosts, and, apart from rare exceptions, the worms remain infertile (*1*–*5*). Infections in humans usually manifest as a single subcutaneous nodule, which is caused by a macrofilaria that is trapped by the immune system (*1*,*6*). Subcutaneous migration of the worm may result in local swellings with changing localization (creeping eruption). In addition, rare cases of organ manifestation have been reported, affecting the lung, male genitals, female breast, or the eye. The latter is found in particular during the migratory phase of the parasite (*1*,*5*–*8*). Because typically only a single worm is present, removal of the parasite from the skin is usually sufficient to treat human infections. Final diagnosis is established by microscopic examination of the excised worm (*5*,*6*). Making a definite species diagnosis on morphologic grounds is difficult, because a large number of zoonotic *Dirofilaria* species have been described that share morphologic features with *D. repens.* Further species probably await description. Here, we report an unusual *D. repens* infection in a resident of Germany who returned from travel to India and Sri Lanka with a subcutaneous nodule containing a gravid female worm and concomitant meningoencephalitis. Molecular analysis identified a *D. repens* strain that was different from those found in public databases.

## The Case

Two days after returning from 9 months of travel in southern India and Sri Lanka, a 45-year-old German man sought treatment at a hospital because of acute speech problems. During the previous 5 weeks, the patient had experienced a persistent headache and creeping eruptions of 5–7 cm on the left arm, which moved from the upper arm to the back of the hand. Physical examination found a tender nodule on the left hand, with a diameter of ≈2 cm, as well as signs of aphasia and apraxia. Cranial magnetic resonance imaging (MRI) indicated cortical and subcortical signal changes in the left frontal region, with signs of meningeal inflammation but no signs of acute ischemia, bleeding, or venous occlusions. Laboratory investigations showed increased cerebrospinal fluid (CSF) protein levels and increased CSF cell counts of 1,500/µL with a high proportion of eosinophils (40%), as well as increased blood leukocyte counts of 12,000/µL (9% eosinophils). Serologic testing showed high antibody titers against *Dirofilaria* antigen and moderate titers against *Strongyloides* antigen, but no significant antibody titers were found against other helminth antigens tested, including *Toxocara, Cysticerca, Schistosoma, Fasciola,* or *Paragonimus* species. Antihelminthic treatment with albendazole (400 mg 2×/d) and concomitantly with methyl-prednisolone (20 mg 2×/d) was initiated, and the patient recovered rapidly.

Removal of the nodule 10 days after the initiation of drug therapy and subsequent histologic examination showed eosinophilic infiltrates and sections of a gravid female nematode that contained large numbers of microfilariae with obtuse cephalic ends and a filiform tail without nuclei. The adult worm showed several characteristics resembling those of *D. repens* (*2*,*5*,*9*,*10*) (Figure). The cuticula was 20 µm thick, multilayered, transverse-striated, and contained large numbers of external longitudinal ridges. Cross-sections showed a well-developed musculature of the coelomyarian type and a worm diameter of ≈550µm. To further confirm the diagnosis of *D. repens* infection, DNA of the worm was extracted (*11*) and panfilarial PCR was performed that targeted the mitochondrial 12S rRNA gene (*11*). Sequence analysis of the 509-bp PCR product and comparison with sequences deposited in GenBank showed the organism had the highest similarity of ≈97% to *D. repens* and of 90% to *D. immitis* (data not shown; the sequence has been submitted to the GenBank database with the accession no. GQ292761).

**Figure:**
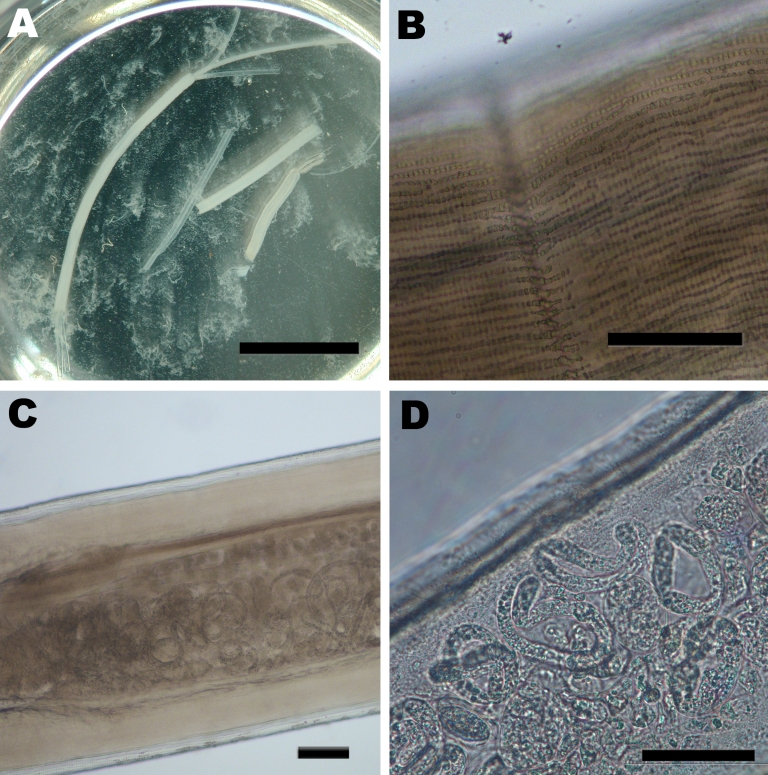
Images of the adult female *Dirofilaria repens* worm removed from a subcutanous nodule of the patient. A) Macroscopic view of sections of the worm in saline in a petri dish. Two uteri and the intestinal tract can be seen protruding from a disrupted end of the largest section. The saline is turbid due to the massive release of microfilariae, which are not discernible at this magnification. Scale bar = 1 cm. B) Microscopic view of the outer cuticula with multiple longitudinal ridges. Scale bar = 100 μm. C) Microscopic view of the worm showing the well-developed muscle layer and the uterus containing microfilariae. Scale bar = 100 μm. D) Higher magnification of a section of the uterus containing multiple microfilariae. Scale bar = 50 µm.

## Conclusion

We report a human *D. repens* infection with concomitant meningoencephalitis. Complications associated with the central nervous system were most likely because of the worm infection as the CSF contained high numbers of eosinophilic granulocytes, the patient recovered rapidly after initiation of antihelminth and anti-inflammatory treatment, and MRI largely excluded other causes such as acute ischemia, bleeding, or venous occlusions. Other helminth infections, in particular, cysticercosis, were unlikely, according to a panel of negative serologic tests. In contrast to other *D. repens* infections in humans, which are usually restricted to the skin, in this case the patient showed blood eosinophilia and high antibody titers to *Dirofilaria* antigen, which indicate a generalized response to the parasite. Moreover, the worm removed from the skin nodule of the patient had developed to maturity and contained microfilariae. These microfilariae were likely responsible for the generalized immune response as well as for the involvement of the central nervous system. Microfilariae that cross the blood-brain barrier and cause neurologic symptoms in humans or animals have been previously described for other species such as *Meningonema peruzzi* and *D. immitis* (*12*,*13*). In the case presented here, infection was most likely acquired in India or Sri Lanka, 2 regions where *D. repens* is endemic (*7*,*8*,*14*,*15*). Notably, genetic analysis of the highly conserved mitochondrial 12S rRNA gene showed a 3% deviation from *D. repens* sequences deposited in public databases, which suggests that different *D. repens* strains vary considerably. Whether specific variants are more likely to develop to maturity and cause generalized disease in humans deserves further investigation.
